# Is it necessary to monitor the serum luteinizing hormone (LH) concentration on the human chorionic gonadotropin (HCG) day among young women during the follicular-phase long protocol? A retrospective cohort study

**DOI:** 10.1186/s12958-022-00888-4

**Published:** 2022-02-02

**Authors:** Wenjuan Zhang, Zhaozhao Liu, Manman Liu, Jiaheng Li, Yichun Guan

**Affiliations:** Reproduction Center, The Third Affiliated Hospital of ZhengZhou University, ZhengZhou, Henan China

**Keywords:** Luteinizing hormone, Pituitary desensitization, Retrieved eggs, Live birth

## Abstract

**Background:**

The normal physiological function of LH requires a certain concentration range, but because of pituitary desensitization, even on the day of HCG, endogenous levels of LH are low in the follicular-phase long protocol. Therefore, our study aimed to determine whether it is necessary to monitor serum LH concentrations on the day of HCG (LH_HCG_) and to determine whether there is an optimal LH_HCG_ range to achieve the desired clinical outcome.

**Methods:**

A retrospective cohort study included 4502 cycles of in vitro fertilization (IVF)/intracytoplasmic sperm injection (ICSI) from January 1, 2016, to June 30, 2019, in a single department. The main outcome measures included retrieved eggs, available embryos, and live birth rate.

**Results:**

The LH_HCG_ was divided into five groups: Group A (LH ≤ 0.5), Group B (0.5 IU/L < LH ≤ 1.2 IU/L), Group C (1.2 IU/L < LH ≤ 2.0 IU/L), Group D (2.0 IU/L < LH ≤ 5.0 IU/L), Group E (LH > 5 IU/L)**.** In terms of the numbers of retrieved eggs (15.22 ± 5.66 vs. 13.54 ± 5.23 vs. 12.90 ± 5.05 vs. 12.30 ± 4.88 vs. 9.6 ± 4.09), diploid fertilized oocytes (9.85 ± 4.70 vs. 8.69 ± 4.41 vs. 8.39 ± 4.33 vs. 7.78 ± 3.96 vs. 5.92 ± 2.78), embryos (7.90 ± 4.48 vs. 6.83 ± 4.03 vs. 6.44 ± 3.88 vs. 6.22 ± 3.62 vs. 4.40 ± 2.55), and high-quality embryos (4.32 ± 3.71 vs. 3.97 ± 3.42 vs. 3.76 ± 3.19 vs. 3.71 ± 3.04 vs. 2.52 ± 2.27), an increase in the LH_HCG_ level showed a trend of a gradual decrease. However, there was no significant difference in clinical outcomes among the groups (66.67% vs. 64.33% vs. 63.21% vs. 64.48% vs. 63.33%). By adjusting for confounding factors, with an increase in LH_HCG_, the number of retrieved eggs decreased (OR: -0.351 95%CI − 0.453-[− 0.249]).

**Conclusion:**

In the follicular-phase long protocol among young women, monitoring LH_HCG_ is recommended in the clinical guidelines to obtain the ideal number of eggs.

**Supplementary Information:**

The online version contains supplementary material available at 10.1186/s12958-022-00888-4.

## Introduction

Appropriate protocols of controlled ovarian stimulation (COS) are critical for assisted reproductive technology (ART) outcomes. The discovery of gonadotropin-releasing hormone (GnRH) analogs has offered multiple options in assisted reproduction and improved in vitro fertilization (IVF) success rates [1]. Effective control of the premature luteinizing hormone (LH) peak, reduction of the cycle cancellation rate and more mature oocytes make the GnRH agonist protocol popular in many reproductive centers. In China, the follicular-phase long protocol has gradually become mainstream because of its simple and convenient single administration, good follicular homogeneity, and excellent fresh cycle outcomes [2]. In the GnRH agonist protocol, because of pituitary desensitization, endogenous levels of LH are very low during the late stimulation phase [3]. Moreover, approximately 50% of patients undergoing IVF/intracytoplasmic sperm injection (ICSI) using a GnRH agonist are LH deficient [3]. Thus, it would seem logical that LH supplementation would be beneficial.

According to the “two-cell, two-gonadotrophin theory”, both follicle-stimulating hormone (FSH) and LH are important for follicle development in humans. This model explains our understanding of folliculogenesis [4]. LH stimulates theca cells, promoting androgen production, and FSH regulates the proliferation of granulosa cells (GCs) and promotes E2 synthesis. Whereas FSH is the main regulator of antral follicular growth, LH plays key roles in promoting steroidogenesis and in the development of the leading follicle. Moreover, LH exerts different functions during the different stages of both natural and stimulated cycles [5]. During the menstrual cycle, LH not only promotes the growth of larger follicles but also increases granulosa cell FSH activity by increasing androgen synthesis, and then LH promotes the recruitment of follicles.

The normal physiological function of LH requires a certain concentration range, namely, the “LH window” [6]. When below the LH threshold, the eggs cannot fully mature, and there is not enough androgen and estrogen synthesis. Moreover, there is a lack of paracrine signals between granulosa cells and membrane cells. In contrast, when beyond the upper limit, granulosa cell proliferation is inhibited, which can lead to a series of problems in egg development. Therefore, we speculate that it is necessary to monitor the level of LH during the early follicular phase long protocol.

An increasing amount of evidence has demonstrated that downregulation with a GnRH agonist in some normogonadotrophic women may result in profound suppression of LH, which in turn impairs adequate estradiol synthesis during FSH stimulation for IVF/ICSI [4–7] and reduces the fertilization rate [8], the number of clinical pregnancies [8], and the pregnancy outcomes [9]. However, there is some controversy about this conclusion.

Consequently, this study aimed to determine whether it is necessary to monitor the serum LH concentration on the HCG day (LH_HCG_), to identify whether the LH_HCG_ has an impact on the clinical outcome and to determine whether there is an optimal LH range to achieve the expected clinical outcome.

## Material and methods

### Study design and participants

**Fig. 1:**
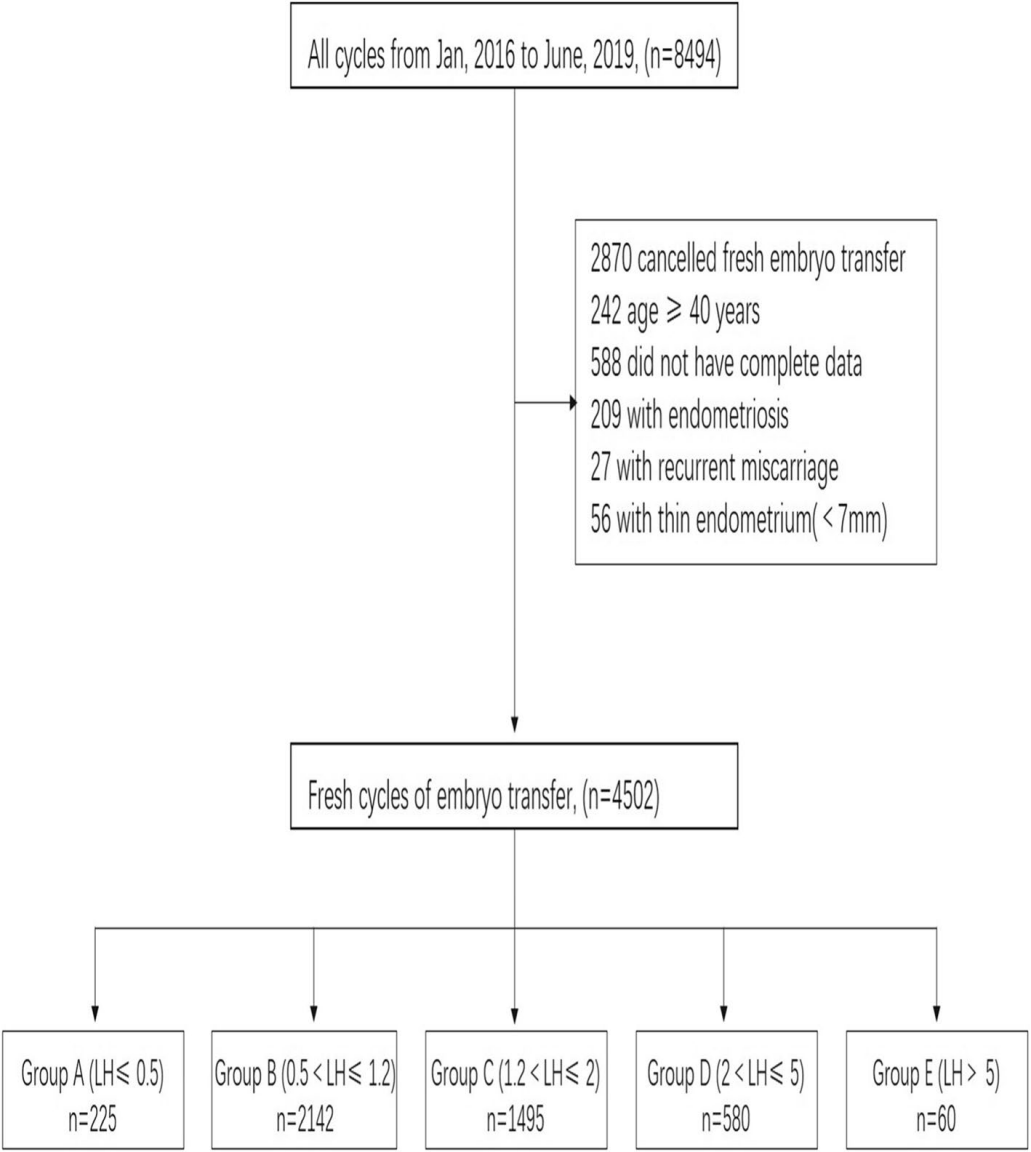
the flow chart of the study

The subjects of this retrospective study underwent 4502 cycles of IVF or ICSI from January 1, 2016, to June 30, 2019, in our department. The following inclusion criteria were applied: 1) age < 40 years, 2) follicular-phase single-dose GnRH agonist protocol, and 3) fresh cycle transplants. Thin endometrium on HCG day, recurrent miscarriage and endometriosis were excluded. Although GnRH-a has a therapeutic effect on endometriosis, patients diagnosed with endometriosis were excluded in this study because endometriosis leads to infertility and affects the outcome of ART outcomes in many ways [10] (Fig. 1).

The study was approved by the ethics committee of the Third Affiliated Hospital of Zhengzhou University (2021–105).

Data on patient age and infertility treatment-related characteristics were collected from the files. Basic sex hormone levels (FSH, LH, estradiol [E2], progesterone [P]), Start-up gonadotropin (Gn) does, Gn dose, number of oocytes retrieved, number of diploid fertilized oocytes, number of embryos, number of embryos transferred, and the LH level on day 1 of ovarian stimulation (HCG_day_1)/the HCG day were recorded. E2/P/on the day of HCG administration was recorded.

### Stimulation protocols

Follicular-phase single-dose GnRH agonist protocol: A single dose of 3.75 mg long-acting GnRH agonist triptorelin (Diphereline, IPSEN, Paris, France) was administered on day 2 or 3 or 4 of the menstrual cycle. Twenty-eight days later, serum FSH, LH, E2, and P were examined. When FSH and LH were both < 5 IU/L, *P* < 3.18 nmol/L and E2 < 183.5 pmol/L, Gn was initiated daily until ovulation induction. Chosing the most appropriate FSH starting dose based on patients’ age, body mass index (BMI), Ant-Mullerian hormone (AMH) and FSH can obtain a more optimized effect [11, 12]. So our FSH starting dose was individualized. Human menopausal gonadotropin (HMG Livzon, China, Zhuhai) or recombinant LH (rLH Luveris Merck Serono S. A, Beijing) was added according to follicular development. When three follicles reached a mean diameter of 17 mm or two follicles reached a mean diameter of 18 mm, 0.25 mg of recombinant HCG (Ovidrel, Merck Serono S.A., Beijing) was administered subcutaneous injection. Oocyte retrieval was performed 36 h–38 h after hCG injection by transvaginal ultrasound-guided single-lumen/double-lumen needle aspiration. ICSI was performed only for severe male factor infertility or previous fertilization failure. Luteal phase support was initiated on day 1 after oocyte retrieval. The cleavage stage embryo transfer time was 3 days after egg retrieval. The blastocyst transfer time was 5 days after egg retrieval. Morphologic criteria were used for embryo scoring [13].

### Primary and secondary outcomes

Biochemical pregnancy was assessed by serum hCG detection 14 days after ET [14]. Clinical pregnancy was confirmed if a fetal heartbeat could be observed by transvaginal ultrasound [14]. Severe ovarian hyperstimulation sydrome (OHSS) was diagnosed in women who were hospitalized because they fulfilled one or more of the following criteria: clinical ascites; hydrothorax; and dyspnea (exertional or at rest) [15]. Early abortion was defined as a clinical pregnancy that failed to reach the 12th gestational week. Live birth was defined as the birth of a live child after 28 weeks of gestation per embryo transfer cycle. Ectopic pregnancy was a pregnancy outside the uterine cavity, diagnosed by ultrasound, surgical visualization or histopathology. The premature birth rate was defined as delivery before 37 gestational weeks but not earlier than 28 gestational weeks [16].

### Statistical analysis

Differences in variables between the groups were statistically analyzed with Student’s t-test and chi-squared tests when appropriate. A bilateral *p* value < 0.05 was considered to be significant. The results are presented as the mean ± standard deviation. If the variances were not uniform, we used the rank sum test for comparison.

Linear regression analysis was performed to assess the association between the LH level on the HCG day and the number of eggs retrieved. The same set of potential confounders was introduced into the regression models for adjustment by the enter method, regardless of whether significant differences between groups were observed. Statistical analysis was performed with the Statistical Package for the Social Sciences (version 24.0; SPSS Inc., USA). All *P* < 0.05 on one-sided tests was considered to be statistically significant.

## Results

When the serum LH level is lower than 0.5 IU/L, both the fertilization rate and the number of embryos decreased significantly. Some clinicians believe that the threshold of LH can be set to 1.2 IU/L; if the LH is lower than this level, follicular development and endometrial growth will be severely insufficient [17]. When the serum LH level is greater than 5.0 IU/L on the day of HCG in the follicular-phase long protocol, the pregnancy rate significantly decreased [18]. Thus, we divided the LH_HCG_ into five groups: Group A (LH ≤ 0.5IU/L), Group B (0.5 IU/L < LH ≤ 1.2 IU/L), Group C (1.2 IU/L < LH ≤ 2.0 IU/L), Group D (2.0 IU/L < LH ≤ 5.0 IU/L), Group E (LH > 5 IU/L).

There were 9 cycles with LH_HCG_ ≥ 10 IU/L in the follicular-phase long protocol, of which only 1 cycle was greater than 20 IU/L. There was no obvious follicle luteinization in these cycles.

According to the basic information and clinical characteristics of the patients, the age of group D was slightly lower than that of group C. The BMI of groups D and E was lower. The basal FSH and LH in groups C, D and E were higher than those in groups A and B (Table 1).

**Table 1:** Patient characteristics of clinical

	Group A (LH ≤ 0.5)	Group B (0.5<LH ≤ 1.2)	Group C (1.2<LH ≤ 2)	Group D (2<LH ≤ 5)	Group E (LH>5)	*P*
No.of cycles	225	2142	1495	580	60	
Age of woman (years)	29.80 ± 3.93	29.69 ± 3.89	29.92 ± 3.82	29.30 ± 3.80^c^	29.65 ± 3.92	0.025
Body mass index (kg/m^2^)	23.91 ± 3.50	23.80 ± 3.22	23.56 ± 3.17	22.49 ± 3.18^abc^	22.52 ± 3.37^ab^	0.000
Basic hormone concentrations						
FSH (IU/L)	6.38 ± 1.86	6.64 ± 1.90	7.06 ± 2.12^ab^	7.35 ± 2.13^ab^	7.73 ± 2.33^ab^	0.000
LH (IU/L)	4.89 ± 3.60	5.38 ± 3.77	5.99 ± 3.90^ab^	6.80 ± 5.67^abc^	6.71 ± 4.81^ab^	0.000
P (nmol/L)	145.60 ± 64.69	152.96 ± 109.13	155.76 ± 146.55	159.43 ± 169.57	164.23 ± 120.90	0.615
E2(pmol/L)	1.52 ± 2.17	1.42 ± 1.79	1.45 ± 3.03^ab^	1.57 ± 3.65	1.90 ± 2.63	0.000
Ant-Mullerian hormone AMH (pmol/L)	37.00 ± 23.12	33.16 ± 22.03	31.90 ± 22.52^a^	32.82 ± 23.50	32.98 ± 23.32	0.029
Start-up Gn does (IU)	163.90 ± 52.36	167.20 ± 56.47	169.17 ± 60.02	157.46 ± 58.74^bc^	157.71 ± 62.91	0.000
Total Gn dose (IU)	2532.36 ± 993.04	2479.22 ± 1012.23	2529.74 ± 1080.28	2351.01 ± 1083.11^abc^	2308.48 ± 1117.57	0.000
LH_day 1_(IU/L)	0.59 ± 0.29	0.72 ± 0.38a	0.97 ± 1.01^ab^	1.06 ± 0.73^abc^	1.05 ± 0.69^ab^	0.000
Hormone concentrations on HCG						
E2(pmol/L)	1367.07 ± 6341.47	12,884.09 ± 5950.26^ad^	13,131.38 ± 5834.56^ad^	14,517.02 ± 6740.33^bc^	13,151.15 ± 6015.93	0.000
P (nmol/L)	3.89 ± 1.42^bcd^	3.56 ± 1.44^cd^	3.35 ± 1.49	3.21 ± 1.42	3.30 ± 1.34	0.000
Endometrial thickness on HCG	11.35 ± 6.85	11.29 ± 2.26	11.41 ± 2.29^ae^	11.40 ± 2.09	10.56 ± 2.01	0.002
No. of follicles(≥14 mm)	13.23 ± 4.47	12.17 ± 4.21^a^	11.61 ± 3.78^ab^	11.36 ± 3.84^ab^	8.75 ± 3.10^abcd^	0.000
No. of follicles(≥16 mm)	9.65 ± 3.47	9.24 ± 3.31	8.94 ± 3.00	8.86 ± 3.16^a^	7.00 ± 2.35^abcd^	0.000
No. of follicles(≥18 mm)	6.11 ± 2.42	6.03 ± 2.20	5.95 ± 2.05	5.99 ± 2.17	4.98 ± 1.94^abcd^	0.000

^a^Compared with group A, *P*<0.05

^b^Compared with group B, *P*<0.05

^c^Compared with group C, *P*<0.05

The level of P in groups A and B was higher than that in the other groups. The AMH value of group C was significantly lower than that of group A. The start-up Gn dose in group D was significantly lower than that in groups B and C. The total Gn dose in group D was significantly lower than that in the other groups. For the hormone concentrations of HCG, the E_2_ levels of group B and group C were lower, and the P level in group A was the highest. On the HCG day, the endometrium in group C was thicker than that in groups A and E (Table 1).

In terms of the numbers of retrieved eggs, embryos, high-quality embryos and diploid fertilized oocytes, an increase in the LH_HCG_ level showed a trend of a gradual decrease. Group E had the highest rate of embryo transfer at the cleavage stage. However, there was no significant difference in clinical outcomes among the groups (Tables 2 and 3).

**Table 2:** Embryological and clinical outcomes

	Group A (LH ≤ 0.5)	Group B (0.5<LH ≤ 1.2)	Group C (1.2<LH ≤ 2)	Group D (2<LH ≤ 5)	Group E (LH>5)	*P*
No.of oocytes	15.22 ± 5.66	13.54 ± 5.23^a^	12.90 ± 5.05^ab^	12.30 ± 4.88^ab^	9.6 ± 4.09^abcd^	0.000
No.of diploid fertilized oocytes	9.85 ± 4.70	8.69 ± 4.41^a^	8.39 ± 4.33^a^	7.78 ± 3.96^ab^	5.92 ± 2.78^abcd^	0.000
No.of embryos	7.90 ± 4.48	6.83 ± 4.03^a^	6.44 ± 3.88^ab^	6.22 ± 3.62^ab^	4.40 ± 2.55^abcd^	0.000
No.of high quality embryos	4.32 ± 3.71	3.97 ± 3.42	3.76 ± 3.19	3.71 ± 3.04	2.52 ± 2.27^abcd^	0.007
No.of embryos transferred	1.58 ± 0.49	1.63 ± 0.48	1.65 ± 0.48	1.66 ± 0.48	1.70 ± 0.46	0.203
Types of embryos transferred						
Cleavage stage embryo rate	62.22%	68.60%	70.03%	70.34%	88.33%^abcd^	0.002
Blastocysts rate	37.78%	31.40%	29.97%	29.66%	11.67%	
OHSS rate	3.56%	2.80%	3.95%	4.66%	6.67%	0.075
Biochemical pregnancy rate	69.33%	67.46%	67.56%	68.62%	66.67%	0.963
Clinical pregnancy rate	66.67%	64.33%	63.21%	64.48%	63.33%	0.868
Ectopic pregnancy rate	2.67%	1.16%	1.59%	1.07%	0	0.469
Early Spontaneous abortion rate	10.67%	9.65%	8.47%	9.09%	2.63%	0.408
Live birth rate	56.44%	55.56%	54.65%	56.03%	60%	0.899
Premature birth rate	21.26%	15.71%	14.44%	17.54%	22.22%	0.216

**Table 3:** Linear regression analysis between serum LH concentration of HCG day and eggs retrieved

	Adjusted OR	95% CI	*P*
LH_HCG_	-0.351	-0.453-(−0.249)	0.000
Age of woman (years)	−0.044	−0.072-(−0.015)	0.003
BMI	0.010	− 0.024-0.045	0.549
Basic LH (IU/L)	−0.030	--0.059-(−0.001)	0.046
AMH	0.004	−0.002-0.009	0.179
Hormone concentrations on HCG			
E2(pmol/L)	0.000	0.000–0.000	0.000
P (nmol/L)	0.297	0.216–0.378	0.000
No. of ≥14 mm folliculars	0.674	0.640–0.707	0.000

**Fig. 2:**
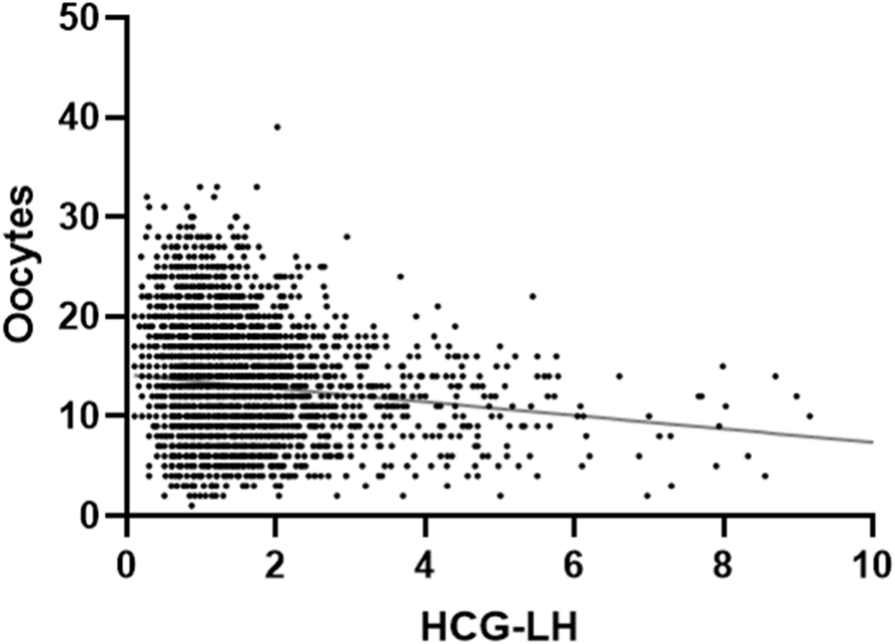
Linear relationship between serum LH concentration of HCG day and eggs retrieved. The figure shows that in the follicular-phase long protocol, with the increase in LH_HCG_, the number of retrieved eggs decreased

Logistic regression analysis was used to determine whether the LH_HCG_ level was related to the number of oocytes retrieved. The results showed that in the follicular-phase long protocol, with the increase in LH_HCG_, the number of retrieved eggs decreased (Table 2 and Fig. 2).

## Discussion

The findings of our study suggest that the level of LH_HCG_ can affect the IVF/ICSI outcome in the follicular-phase long protocol in a young population.

The “two cell–two gonadotropin” model has been the key to our understanding of folliculogenesis [4]. According to this model, LH stimulates theca cells, thereby advancing androgen production, and FSH governs the proliferation of granulosa cells (GCs) and promotes E2 synthesis. Both FSH and LH play an important role in folliculogenesis. The details of the specific role of LH in folliculogenesis have not been fully studied.

LH, which is well known for its importance in the late follicular phase, supports theca cells in the ovary to provide androgens and hormonal precursors for estradiol production. LH not only promotes the growth of larger follicles but also increases FSH activity in granulosa cells by increasing androgen synthesis [19]. During the development of a dominant follicle, its dependence on FSH gradually declines, whereas its dependence on LH gradually increases. FSH’s partial role is replaced by LH in the late follicular phase. This facilitates the selection and maintenance of the dominant follicle.

The follicular-phase long protocol uses a single dose of 3.75 mg GnRH-a in the early follicular phase for pituitary downregulation. Two weeks after a single injection of 3.75 mg GnRH-a, endogenous sex hormones were almost completely inhibited. The FSH levels gradually recover after 3–4 weeks, and the E_2_ levels begin to rise after 7–8 weeks, whereas the inhibitory effect on LH lasts up to 8 weeks after administration [20]. After 30 days, as the FSH level gradually recovers, Gn is used to stimulate the development of multiple follicles in the ovary. In the follicular-phase long protocol, due to pituitary desensitization, endogenous LH levels are very low in the later stage of stimulation. Previous studies had shown that when the serum LH concentration after pituitary downregulation is between 0.5–2.5 U/L, we can obtain a normal ovarian response [21]. Too high or too low is not ideal.

Physiological levels of LH are clearly important for follicle development. If the LH level is abnormal, it will lead to abnormal follicular development. Therefore, it was speculated that there should be an LH threshold window. The Asia-Pacific consensus on the application of LH in assisted reproductive treatment published in 2011 concluded that the reasonable threshold window for LH ranges from 1.2–5 IU/L [22]. However, some studies found that in the process of controlled ovarian hyperstimulation (COH), when the LH level was lower than 1.2 IU/L on the HCG day, it did not affect the clinical outcome [23], whereas for the follicular-phase long protocol, LH was very rarely elevated due to suppression. In this study, the LH concentration was greater than 10 IU/L in only 10 cases, there was only 1 case with LH > 20 IU/L, and the P concentrations on the HCG day were all normal.

Our study found that in the follicular-phase long protocol, with an LH ≤ 0.5 IU/L on the hCG day, patients had a higher number of oocytes retrieved, a better embryo rate, and more embryos, but there was no significant difference in the live birth rate compared with the other groups. Thus, we speculate that when the LH ≤0.5 IU/L, although there may be pituitary hypersuppression leading to inadequate LH secretion in the late follicular phase, it does not affect the outcome. This is similar to the results of the study of Brjercke et al. [24], that is, a lower LH can satisfy the development of follicles. As long as less than 1% of LH receptors are occupied, they can play a normal physiological role [24]. However, when there are older women, a low response, a slow response, etc. in the COH cycle, adding LH may be beneficial. With a normal ovarian response, a low LH concentration does not affect the fertilization outcome [25].

However, as LH increased, the numbers of oocytes retrieved, embryos, and high-quality embryos and the number of diploid fertilized oocytes decreased gradually, which may be because excessive LH levels may cause the ovaries to secrete more androgens, resulting in follicular atresia and then fewer oocytes retrieved. However, the pregnancy rate did not decrease, which may be because the increase in LH level only affected the number of oocytes retrieved but did not affect the quality of the oocytes, the embryo development potential or endometrial receptivity. For Preimplantation Genetic Testing (PGT) patients, it may be more suitable to control the LH at a lower level on the HCG day to retrieve more oocytes.

The main limitations of this article are as follows: 1. The current measurement of LH levels may not accurately reflect the true level of internal LH; 2. Our study mainly enrolled young people, which is a specific group. This may have a certain influence on the outcome; 3. According to follicular development in the later stage, HMG or rLH may be added, which may cause the conclusion to be inaccurate. The cumulative pregnancy rate and cumulative live birth rate were not calculated. The data sample is still not large enough, and additional prospective large-sample studies are needed to confirm this idea.

In conclusion, in the follicular-phase long protocol, the LH level is maintained at a low level throughout COH. From the data of our study, we should pay more attention to LH_HCG_. Elevated LH levels on the hCG day may lead to a reduction in oocyte retrieval. However, even when LH ≥ 5 IU/L, approximately 10 oocytes are retrieved, which, for clinical purposes, is sufficient. However, those who undergo PGT may benefit more when the LH level is controlled within a certain range. Therefore, in the follicular-phase long protocol among young women, monitoring LH_HCG_ has clinical applications.

## Supplementary Information

**Additional file 1.**

## Data Availability

The data sets used and/or analyzed during the current study are available from the corresponding author on reasonable request.

## References

[1] Shrestha D, La X, Feng HL (2015). Comparison of different stimulation protocols used in in vitro fertilization: a review. Ann Transl Med.

[2] Liu S, Deng C (2018). Is LH supplementation necessary in the follicular-phase long protocol?. J Reprod Med.

[3] Westergaard LG, Laursen SB, Andersen CY (2000). Increased risk of early pregnancy loss by profound suppression of luteinizing hormone during ovarian stimulation in normogonadotrophic women undergoing assisted reproduction. Hum Reprod.

[4] Falck B (1959). Site of production of oestrogen in rat ovary as studied in micro-transplants. Acta Physiol Scand Suppl.

[5] Fritz MA, Speroff L (2011). Clinical gynecologic endocrinology and infertility.

[6] Balasch J, Fábregues F (2002). Is luteinizing hormone needed for optimal ovulation induction?. Curr Opin Obstet Gynecol.

[7] Westergaard LG, Erb K, Laursen S, Rasmussen PE, Rex S (1996). The effect of human menopausal gonadotrophin and highly purified, urine-derived follicle stimulating hormone on the outcome of in-vitro fertilization in down-regulated normogonadotrophic women. Hum Reprod.

[8] Fleming R, Lloyd F, Herbert M, Fenwick J, Griffiths T, Murdoch A (1998). Effects of profound suppression of luteinizing hormone during ovarian stimulation on follicular activity, oocyte and embryo function in cycles stimulated with purified follicle stimulating hormone. Hum Reprod.

[9] Fleming R, Rehka P, Deshpande N, Jamieson ME, Yates RW, Lyall H (2000). Suppression of LH during ovarian stimulation: effects differ in cycles stimulated with purified urinary FSH and recombinant FSH. Hum Reprod.

[10] Terzic M, Aimagambetova G, Garzon S (2020). Ovulation induction in infertile women with endometriotic ovarian cysts: Current evidence and potential pitfalls [J]. Minerva Med..

[11] Di Paola R, Garzon S, Giuliani S (2018). Are we choosing the correct FSH starting dose during controlled ovarian stimulation for intrauterine insemination cycles? Potential application of a nomogram based on woman's age and markers of ovarian reserve [J]. Arch Gynecol Obstet..

[12] Burnik Papler T, Vrtacnik Bokal E, Prosenc Zmrzljak U. PGR and PTX3 gene expression in cumulus cells from obese and normal weighting women after administration of long-acting recombinant follicle-stimulating hormone for controlled ovarian stimulation [J]. Arch Gynecol Obstetr. 2019;299(3):863–71. 10.1007/s00404-018-5031-y 30607593

[13] Nasiri N, Eftekhari-Yazdi P (2015). An overview of the available methods for morphological scoring of pre-implantation embryos in in vitro fertilization. Cell J.

[14] Fernando Z (2017). G. David a, Slike D. the international glossary on infertility and fertility care. Fertil Steril.

[15] Navot D, Bergh PA, Laufer N (1992). Ovarian hyperstimulation syndrome in novel reproductive technologies: prevention and treatment. Fertil Steril.

[16] National Collaborating Centre for Women’s and Children’s Health (UK) (2015). Preterm labour and birth.

[17] Liang X (2018). Clinical practice and improvement of assisted reproductive technology.

[18] Wu XX, Chen DL, Zheng YP (2019). The effect of LH concentration on hCG day on the outcome of in vitro fertilization in the follicular-phase long protocol. J Huazhong Univ Sci Technol (Health Sci).

[19] Sungurtekin U, Jansen RP (1995). Profound luteinizing hormone suppression after stopping the gonadotropin-releasing hormone-agonist leuprolide acetate. Fertil Steril.

[20] Broekmans FJ, Bernardus RE, Berkhout G, Schoemaker J (1992). Pituitary and ovarian suppression after early follicular and mid-luteal administration of a LHRH agonist in a depot formulation: decapeptyl CR. Gynecol Endocrinol.

[21] Alviggi C, Clarizia R, Mollo A, Ranieri A, De Placido G (2006). Outlook: who needs LH in ovarian stimulation?. Reprod BioMed Online.

[22] Wong PC, Qiao J, Ho C, Ramaraju GA, Wiweko B, Takehara Y (2011). Current opinion on use of luteinizing hormone supplementation in assisted reproduction therapy: an Asian perspective. Reprod BioMed Online.

[23] Lossl K, Andersen AN, Loft A, Freiesleben NL, Bangsbøll S, Andersen CY (2006). Androgen priming using aromatase inhibitor and hCG during early-follicular-phase GnRH antagonist down-regulation in modified antagonist protocols. Hum Reprod.

[24] Bjercke S, Fedorcsak P, Abyholm T, Storeng R, Ertzeid G, Oldereid N (2005). IVF/ICSI outcome and serum LH concentration on day 1 of ovarian stimulation with recombinant FSH under pituitary suppression. Hum Reprod.

[25] Alviggi C, Conforti A, Esteves SC, Andersen CY, Bosch E, Bühler K (2018). Recombinant luteinizing hormone supplementation in assisted reproductive technology: a systematic review. Fertil Steril.

