# Supplementary material for: Is it necessary to monitor the serum luteinizing hormone (LH) concentration on the human chorionic gonadotropin (HCG) day among young women during the follicular-phase long protocol? A retrospective cohort study
Source: Reprod Biol Endocrinol. 2022 Feb 2;20:24. doi: 10.1186/s12958-022-00888-4 (PMC8808976; doi:10.1186/s12958-022-00888-4)
Supplement: Supplementary file 1 — Additional file 1. [file 12958_2022_888_MOESM1_ESM.docx]

Table 4.Logistic regression analysis between serum LH concentration of HCG day and the incidence of serious OHSS.

|  | Adjusted OR | 95% CI | *P* |
| --- | --- | --- | --- |
| LH_HCG_ | 1.123 | 1.018-1.238 | 0.020 |
| Age of woman(years) | 0.998 | 0.948-1.051 | 0.950 |
| BMI | 0.918 | 0.864-0.976 | 0.005 |
| Basic FSH (IU/L) | 0.984 | 0.897-1.080 | 0.740 |
| AMH | 1.003 | 0.995-1.011 | 0.491 |
| No. of antral follicules | 1.046 | 1.012-1.082 | 0.009 |
| Start-up Gn does（IU） | 0.997 | 0.991-1.003 | 0.355 |
| Total Gn dose (IU) | 1.000 | 0.999-1.000 | 0.039 |
